# Sandwiched Cathodes Assembled from CoS_2_‐Modified Carbon Clothes for High‐Performance Lithium‐Sulfur Batteries

**DOI:** 10.1002/advs.202101019

**Published:** 2021-06-02

**Authors:** Jun Xu, Likun Yang, Shoufu Cao, Jingwen Wang, Yuanming Ma, Junjun Zhang, Xiaoqing Lu

**Affiliations:** ^1^ School of Microelectronics Hefei University of Technology Hefei 230009 P. R. China; ^2^ School of Materials Science and Engineering China University of Petroleum Qingdao Shandong 266580 P. R. China; ^3^ School of Physics and Materials Engineering Hefei Normal University Hefei 230601 P.R. China

**Keywords:** carbon clothes, catalysts, CoS_2_, lithium‐sulfur batteries, sandwiched cathodes

## Abstract

Structural design of advanced cathodes is a promising strategy to suppress the shuttle effect for lithium‐sulfur batteries (LSBs). In this work, the carbon cloth covered with CoS_2_ nanoparticles (CC‐CoS_2_) is prepared to function as both three‐dimensional (3D) current collector and physicochemical barrier to retard migration of soluble lithium polysulfides. On the one hand, the CC‐CoS_2_ film works as a robust 3D current collector and host with high conductivity, high sulfur loading, and high capability of capturing polysulfides. On the other hand, the 3D porous CC‐CoS_2_ film serves as a multifunctional interlayer that exhibits efficient physical blocking, strong chemisorption, and fast catalytic redox reaction kinetics toward soluble polysulfides. Consequently, the Al@S/AB@CC‐CoS_2_ cell with a sulfur loading of 1.2 mg cm^−2^ exhibits a high rate capability (≈823 mAh g^−1^ at 4 C) and delivers excellent capacity retention (a decay of ≈0.021% per cycle for 1000 cycles at 4 C). Moreover, the sandwiched cathode of CC‐CoS_2_@S/AB@CC‐CoS_2_ is designed for high sulfur loading LSBs. The CC‐CoS_2_@S/AB@CC‐CoS_2_ cells with sulfur loadings of 4.2 and 6.1 mg cm^−2^ deliver high reversible capacities of 1106 and 885 mAh g^−1^, respectively, after 100 cycles at 0.2 C. The outstanding electrochemical performance is attributed to the sandwiched structure with active catalytic component.

## Graphical abstract

Sandwiched cathodes constructed from CoS_2_‐modified carbon clothes (CC‐CoS_2_) are designed for high sulfur loading lithium‐sulfur batteries. The sandwiched cathode not only offers three‐dimensional (3D) current collector and host with enough voids for volume expansion to maintain the structural stability, but also promotes the physical encapsulation, chemical entrapment and catalytic conversion of polysulfides species to suppress the shuttle effect.

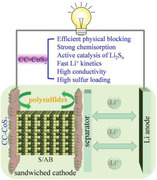

## 1 Introduction

To meet the requirement of developing rechargeable batteries with high energy density and long cycle life, lithium‐sulfur batteries (LSBs) are regarded as one of the distinguished devices for next generation rechargeable batteries, owing to the natural abundance, low cost, high theoretical specific capacity of 1675 mAh g^−1^ (five times the capacity of LiCoO_2_), high gravimetric energy density of 2600 Wh kg^−1^ and high volumetric energy density of 2800 Wh L^−1^ of sulfur cathode.^[^ ^1^, ^2^, ^3^, ^4^ ^]^ However, the LSBs suffer from a series of intrinsic problems: 1) the severe “shuttle effect” caused by dissolution of intermediate lithium polysulfide (Li_2_S*_n_*, 4 ≤ n ≤ 8) species into electrolyte during the discharge/charge process, leading to a dramatic capacity loss and a low coulombic efficiency,^[^ ^5^, ^6^, ^7^, ^8^ ^]^ 2) the insulating nature of both sulfur (the active material, 10^−30^ S cm^−1^) and Li_2_S (the product after discharge, 10^−14^ S cm^−1^),^[^ ^9^, ^10^, ^11^ ^]^ and 3) the huge volumetric expansion of sulfur (≈80%) upon lithiation owing to the density difference between sulfur (2.03 g cm^−3^) and Li_2_S (1.66 g cm^−3^).^[^ ^12^, ^13^, ^14^ ^]^ These issues become more serious in high sulfur loading LSBs. Moreover, some additional problems may occur: the thick active material film on the planar Al foil current collector is prone to crack and fall off during discharge/charge process;^[^ ^15^ ^]^ and the increasing thickness of active material layer blocks the Li^+^ transport and increases the resistance, leading to deteriorated electrochemical performance.^[^ ^16^ ^]^

To overcome the above‐mentioned issues and improve the electrochemical performance of LSBs, many efforts have been devoted to developing advanced sulfur hosts and multifunctional separators.^[^ ^17^, ^18^, ^19^, ^20^, ^21^, ^22^, ^23^ ^]^ The carbonaceous materials, such as mesoporous carbon (CMK‐3),^[^ ^24^, ^25^ ^]^ reduced graphene oxide,^[^ ^26^, ^27^ ^]^ carbon nanotubes,^[^ ^28^, ^29^ ^]^ multichannel fibers,^[^ ^30^, ^31^, ^32^ ^]^ were adopted as advanced sulfur hosts to reduce the capacity fading. These carbon‐based host materials typically have essential characteristics of high electronic conductivity, large specific surface area, and porous structures, which can improve the conductivity, realize high sulfur loading and reduce the influence of volumetric expansion. However, the LSBs assembled from such carbonaceous hosts still suffer from poor rate capability and cycling performance, as the physical adsorption between the nonpolar carbonaceous materials and soluble polysulfides is usually insufficient to restrain the shuttle effect, inevitably causing low sulfur utilization.^[^ ^33^, ^34^ ^]^ On the other hand, some polar metal compounds, such as metal organic frameworks,^[^ ^35^, ^36^, ^37^ ^]^ metal oxides,^[^ ^38^, ^39^, ^40^ ^]^ metal sulfides,^[^ ^41^, ^42^, ^43^ ^]^ metal nitrides,^[^ ^44^, ^45^ ^]^ exhibit strong chemisorption of soluble polysulfides and are capable of capturing polysulfides species. Meanwhile, they can also function as an active catalyst which plays a key role in catalyzing polysulfide redox reactions (conversion of soluble Li_2_S*_n_* to Li_2_S_2_/Li_2_S) and accelerating the reaction kinetics.^[^ ^43^ ^]^ Shuttling of polysulfide species can be effectively retarded when these polar metal compounds are introduced in the host and/or the separator.^[^ ^39^, ^41^, ^42^ ^]^ For example, cobalt sulfides have intensively investigated in host/interlayer/separator of LSBs. They can effectively capture polysulfides and promote their reversible redox kinetics, thereby resulting in improved electrochemical performance.^[^ ^46^, ^47^, ^48^, ^49^, ^50^, ^51^, ^52^, ^53^ ^]^ Therefore, designing advanced cell configuration to integrate both physical confinement and chemical adsorption of polysulfides is a promising route to enhance electrochemical performance of LSBs, especially the high sulfur loading LSBs.

Herein, we design and fabricate advanced cathodes constructed from CoS_2_ nanoparticles coated carbon cloth (CC‐CoS_2_) for high‐performance LSBs. The free‐standing CC‐CoS_2_ can serve as a robust and flexible three‐dimensional (3D) current collector with a good conductivity for fast electron transport, and the 3D structure with sufficient void space can not only increase the sulfur loading but also effectively buffer the volume expansion during the lithiation/delithiation process. On the other hand, owing to the small size and excellent polarity of the uniformly covered CoS_2_ nanoparticles, the CC‐CoS_2_ provides strong localized chemical binding with lithium polysulfides and benefits for fast interfacial charge transfer. Therefore, the CC‐CoS_2_ film can be adopted as an efficient permselective barrier that realizes multiple functions of efficient physical blocking, strong chemical adsorption, and rapid catalytic conversion of lithium polysulfides, as well as fast lithium‐ion transport kinetics. As a result, the Al@S/AB@CC‐CoS_2_ cell with low sulfur loading of ≈1.2 mg cm^−2^ exhibits high rate capability and excellent capacity retention with a low decay of ≈0.021% per cycle for 1000 cycles at 4 C. Moreover, the sandwiched cathodes assembled from CC‐CoS_2_@S/AB@CC‐CoS_2_ are also designed and fabricated for high sulfur loading LSBs (3.0–6.1 mg cm^−2^) with significantly improved electrochemical performance.

## 2 Results and Discussion

**Figure 1:**
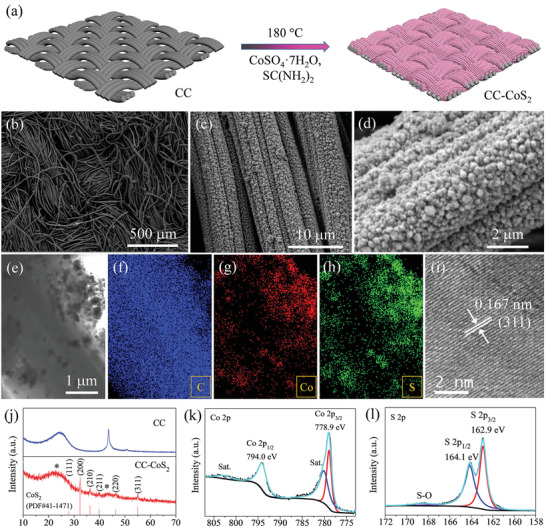
a) Schematic illustration for the synthesis of CC‐CoS_2_. b–d) SEM images of CC‐CoS_2_. e–h) TEM image and corresponding EDX mappings of CC‐CoS_2_. i) HRTEM image of the CoS_2_ nanoparticle. j) XRD patterns of bare CC and CC‐CoS_2_. k,l) Co 2p and S 2p core‐level XPS spectra of CC‐CoS_2_.

The CC‐CoS_2_ was prepared by a simple one‐pot hydrothermal synthesis of CoS_2_ nanoparticles on a CC scaffold using CoSO_4_·7H_2_O and SC(NH_2_)_2_ as the reactants, as shown in **Figure** **1**a. Figure 1b–d shows typical scanning electron microscopy (SEM) images of the as‐prepared CC‐CoS_2_, which can well reserve 3D morphology of the bare CC built from carbon fibers (Figure S1, Supporting Information). However, the surfaces of the fibers become much rough and are uniformly covered by small CoS_2_ nanoparticles with several tens of nanometers. Figure 1e displays a transmission electron microscopy (TEM) image of the CC‐CoS_2_ fiber, and corresponding energy dispersive X‐ray spectroscopy (EDX) mappings of C, Co, S are shown in Figure 1f–h, respectively, which reveals that the CoS_2_ nanoparticles are covered on surface of carbon fiber. Figure 1i presents a high‐resolution TEM (HRTEM) image of the CoS_2_ nanoparticle. The lattice fringes distance of 0.167 nm matches well with the (311) interplanar spacing of cubic CoS_2_, confirming formation of crystalline CoS_2_ nanoparticles. X‐ray diffraction (XRD) pattern of CC‐CoS_2_ is shown in Figure 1j. All diffraction peaks can be well indexed to those of CoS_2_ (PDF#41‐1471), except the two peaks marked with “*” corresponding to CC. The Raman spectrum in Figure S2, Supporting Information, has two resonance peaks at 290 and 390 cm^−1^, which agree with the previously reported values of CoS_2_.^[^ ^54^ ^]^ X‐ray photoelectron spectroscopy (XPS) was also performed to further investigate the surface chemical composition of CoS_2_. Figure 1k shows the Co 2p region, where there are two prominent peaks at 778.9 and 794.0 eV corresponding to Co 2p_3/2_ and Co 2p_1/2_ of Co^2+^, respectively.^[^ ^55^, ^56^ ^]^ The appearance of satellite peaks at 780.1 and 802.6 eV also supports the presence of Co^2+^.^[^ ^57^ ^]^ For the S 2p region in Figure 1l, a couple of peaks at 162.9 and 164.1 eV can be assigned to S 2p_3/2_ and S 2p_1/2_,^[^ ^58^ ^]^ and a minor peak at 168.8 eV can be assigned to the sulfate impurity.^[^ ^59^ ^]^ Therefore, the above results demonstrate the formation of CoS_2_ nanoparticles on CC surface.

**Figure 2:**
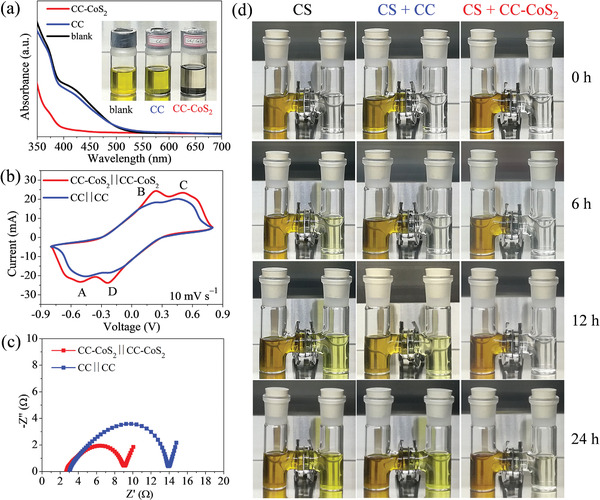
a) UV–vis spectra of the Li_2_S_6_ in DOL/DME solutions before and after exposure to CC and CC‐CoS_2_ for 12 h, respectively; inset: the photograph of the three solutions. b) CV curves of the symmetric cells assembled from CC‐CoS_2_ and CC, respectively, in 0.2 m Li_2_S_6_ electrolyte scanned at 10 mV s^−1^. c) Nyquist plots of the symmetric cells assembled from CC‐CoS_2_ and CC, respectively. d) Photographs of H‐type glass cell filled with the lithium polysulfide (Li_2_S_6_) in DOL/DME solution (the left chamber) and the bare DOL/DME solvent (the right chamber), and separated by the bare Celgard separator (i.e., CS), the separator with a CC film (i.e., CS+CC), and the separator with a CC‐CoS_2_ film (i.e., CS+CC‐CoS_2_) taken after testing durations of 0, 6, 12, and 24 h.

To evaluate chemical adsorption capability of the CC‐CoS_2_ and bare CC for lithium polysulfides, visual adsorption tests were carried out. As shown inset of **Figure** **2**a, the blank Li_2_S_6_ solution has a brown color. When the CC was immersed in the Li_2_S_6_ solution for 24 h, the color keeps unchanged, which indicates the weak adsorption capability of the nonpolar CC. It is worth noting that the Li_2_S_6_ solution after treatment with CC‐CoS_2_ for 24 h becomes almost transparent, thereby indicating the outstanding adsorption capability of CC‐CoS_2_ owing to the large polarity of CoS_2_. More precise analysis was achieved by the UV–vis spectroscopy. The blank Li_2_S_6_ solution has an obvious absorption shoulder at 400–500 nm.^[^ ^60^ ^]^ The peak intensity is only slightly decreased for the Li_2_S_6_ solution treated with CC, however, the peak almost disappears for the Li_2_S_6_ solution treated with CC‐CoS_2_. This observation is in good agreement with visual adsorption test, indicating the strong adsorption capability of CC‐CoS_2_.

Symmetrical cells constructed by two identical CC‐CoS_2_ (or CC) as electrodes and filled with the electrolyte of 0.2 m Li_2_S_6_ in 1,3‐dioxolane (DOL) and dimethyl (DME) were fabricated to study catalytic activity of CC‐CoS_2_ (or CC) on polysulfide redox kinetics. Cyclic voltammetry (CV) curves of the symmetric cells of CC‐CoS_2_||CC‐CoS_2_ and CC||CC are shown in Figure 2b. The CV curves exhibit four pronounces reduction/oxidation peaks, which can be assigned to the electrochemical redox reactions of polysulfides on the electrodes: Peak A arises from the reduction of Li_2_S_6_ → Li_2_S; Peak B is the oxidation of Li_2_S→ Li_2_S_6_; Peak C is the oxidation of Li_2_S_6_ → Li_2_S_8_ and Peak D can be attributed to the reduction of Li_2_S_8_ → Li_2_S_6_.^[^ ^61^ ^]^ The redox peak current is larger in the CC‐CoS_2_||CC‐CoS_2_ cell than that of the CC||CC one. This result suggests that CC‐CoS_2_ has a better catalytic activity than CC. The Nyquist plots of the two symmetric cells are shown is Figure 2c. Compared with the CC||CC cell, the CC‐CoS_2_||CC‐CoS_2_ cell exhibits a much smaller semicircle in the high frequency range, revealing a significantly reduced charge transfer resistance (*R*_ct_) at the electrode‐electrolyte interface. It indicates that the CoS_2_ nanoparticles covered on the CC surface can serve as an active electrocatalyst and accelerate the conversion kinetics of polysulfides.

Blocking of polysulfide diffusion by the CC‐CoS_2_ and CC films were carried out in an H‐type glass, where the left chamber contains the Li_2_S_6_ in DOL/DME solution, and the right chamber is the transparent DOL/DME solution. The Celgard separator (i.e., CS), the Celgard separator with CC (i.e., CS+CC) and the Celgard separator with CC‐CoS_2_ (i.e., CS+CC‐CoS_2_) were employed in the middle of the two chambers, respectively. We took pictures and recorded color changes of the solutions after resting for different time (Figure 2d). As an increase in the resting time (0–24 h), the color of the right chamber solution gradually changes from transparent to yellow for both CS and CS+CC, indicating more polysulfides were diffused in. After resting for 24 h, however, the right chamber solution shows little change for CS+CC‐CoS_2_. Such an observation reveals CC‐CoS_2_ film can effectively retard the polysulfide diffusion and suppress the shuttle effect.

**Figure 3:**
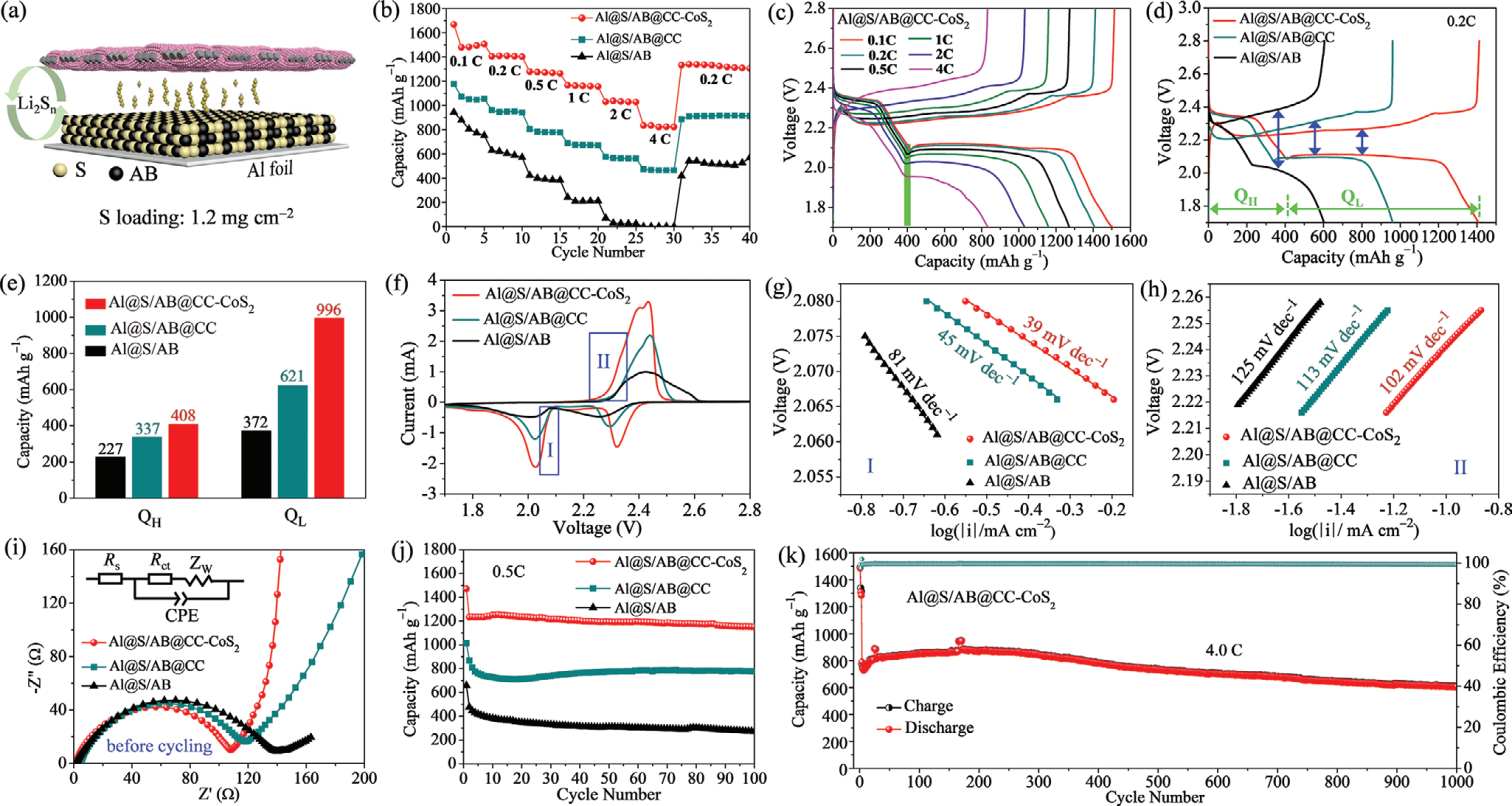
a) Schematic illustration of the Al@S/AB@CC‐CoS_2_ cell. b) Rate performance of the three cells: Al@S/AB@CC‐CoS_2_, Al@S/AB@CC, and Al@S/AB. c) Galvanostatic discharge/charge profiles of Al@S/AB@CC‐CoS_2_ cell at current density varying from 0.1 to 4 C. d) Galvanostatic discharge/charge profiles of the three cells at 0.2 C. e) The discharge capacities of Al@S/AB@CC‐CoS_2_, Al@S/AB@CC, and Al@S/AB cell (Q_H_ and Q_L_ represent the high and low discharge plateaus capacities). f) CV curves of the Al@S/AB@CC‐CoS_2_, Al@S/AB@CC, and Al@S/AB cells scanned at 0.1 mV s^−1^. g, h) The corresponding Tafel plots obtained from CV curves in panel f). i) Nyquist plots of the three cells before cycling, inset is the corresponding equivalent circuit. j) Cycling performance of the three cells at 0.5 C. k) Long‐term cycling performance of the Al@S/AB@CC‐CoS_2_ cell at 4.0 C.

In order to investigate effect of CC‐CoS_2_ film as a barrier layer on improving electrochemical performance, LSBs without and with CC‐CoS_2_ and CC interlayer were fabricated by coating the active material (S) mixed with acetylene black (AB) on the traditional current collector of Al foils. The sulfur loading density on the Al foils is ≈1.2 mg cm^−2^. **Figure** **3**a shows a schematic diagram of the Al@S/AB@CC‐CoS_2_ cathode, where the CC‐CoS_2_ film was covered on the surface of the active material layer. The rate capabilities of the Al@S/AB@CC‐CoS_2_, Al@S/AB@CC and Al@S/AB cells were evaluated by cycling under different current rates (0.1–4 C) (Figure 3b). The Al@S/AB cell delivers low discharge capacities of 805, 603, 390, 208, 26, and 1 mAh g^−1^ at the current rates of 0.1, 0.2, 0.5, 1, 2, and 4 C, respectively. Compared with the Al@S/AB cell, both the Al@S/AB@CC‐CoS_2_ and Al@S/AB@CC cells show significantly improved specific capacities in the wide current range (0.1–4 C), and the Al@S/AB@CC‐CoS_2_ cell exhibits the best rate performance. The reversible specific capacities of the Al@S/AB@CC‐CoS_2_ cell are as high as 1481, 1408, 1271, 1159, 1031, and 823 mAh g^−1^ at the current rates set for 0.1, 0.2, 0.5, 1, 2, and 4 C, respectively. The capacity recovers to 1333 mAh g^−1^ when the current rate decreases from 4 to 0.2 C. Figure 3c shows the galvanostatic charge/discharge profiles of Al@S/AB@CC‐CoS_2_ from 0.1 to 4 C in the potential range of 1.7–2.8 V. There are two obvious reaction plateaus along with sloping regions in the various discharge curves. The plateau at ≈2.3 V is attributed to the conversion of S_8_ to Li_2_S*_n_* (4 ≤ n ≤ 8), and the lower plateau at ≈2.1 V is assigned to the reaction process of Li_2_S_4_ to Li_2_S_2_/Li_2_S.^[^ ^11^ ^]^ Q_H_ represents the discharge capacities at the high discharge voltage plateaus (≈2.3 V), and Q_L_ represents the discharge capacities of the low voltage plateaus (≈2.1 V).^[^ ^59^ ^]^ As discharge current increases from 0.1 to 4 C, the large capacity loss (658 mAh g^−1^) of Al@S/AB@CC‐CoS_2_ is mainly attributed to Q_L_ loss, while Q_H_ has little decay (24 mAh g^−1^). Importantly, the two distinct discharge voltage plateaus for Al@S/AB@CC‐CoS_2_ cell can still be observed at high rate of 4 C, implying excellent electrochemical dynamic behavior. In contrast, the two discharge voltage plateaus at 4 C almost disappear for the Al@S/AB@CC cell (Figure S3, Supporting Information). Theoretically, Q_H_ and Q_L_ are 419 and 1256 mAh g^−1^, respectively, corresponding to Q_L_/Q_H_ = 3.0.^[^ ^59^ ^]^ The Q_L_/Q_H_ ratio reflects the capability of converting polysulfides.^[^ ^62^ ^]^ Figure 3d displays charge/discharge profiles of Al@S/AB@CC‐CoS_2_, Al@S/AB@CC and Al@S/AB cells at a current density of 0.2 C. The Q_H_ and Q_L_ values of the three cells are calculated and presented in Figure 3e. The Al@S/AB@CC‐CoS_2_ cell has a Q_L_/Q_H_ ratio of 2.44, which is higher than those of Al@S/AB@CC cell (1.84) and Al@S/AB cell (1.63). It indicates the significantly promoted conversion of Li_2_S*_n_* (4 ≤ n ≤ 8) to Li_2_S_2_/Li_2_S in the Al@S/AB@CC‐CoS_2_ cell. Furthermore, the charge/discharge profiles of Al@S/AB@CC‐CoS_2_ cell exhibit less polarization than those of Al@S/AB@CC and Al@S/AB cells.

Figure 3f shows CV curves of the Al@S/AB@CC‐CoS_2_, Al@S/AB@CC, and Al@S/AB cells scanned at 0.1 mV s^−1^. There are two cathodic peaks and two overlapped anodic peaks. In comparison with the Al@S/AB@CC and Al@S/AB cells, the Al@S/AB@CC‐CoS_2_ cell delivers higher current density and less polarization of the cathodic/anodic peaks, indicating its accelerated redox reaction kinetics. In order to further analyze the catalytic effects of the cathode reactions, both cathodic and anodic Tafel plots derived from the polarization curves (regions I and II in Figure 3f) are shown in Figure 3g,h, respectively. The Tafel slope at cathodic sweep is 39 mV dec^−1^ for Al@S/AB@CC‐CoS_2_, 45 mV dec^−1^ for Al@S/AB@CC and 81 mV dec^−1^ for Al@S/AB (Figure 3g). For the anodic sweep, the Al@S/AB@CC‐CoS_2_ also has the smallest Tafel slope. The lower Tafel slope reveals the better catalytic activity.^[^ ^63^ ^]^ Therefore, Al@S/AB@CC‐CoS_2_ exhibits the highest catalytic activity to accelerate the polysulfide conversion reactions.

To get a deep understanding of the improved electrochemical performance, electrochemical impedance spectroscopy (EIS) of the cells before and after cycling was performed. The Nyquist plots of the three cells before cycling are shown in Figure 3i. Each plot consists of a single semicircle in the high to medium frequency region and an inclined line at low frequency, which can be ascribed to the charge transfer resistance (*R*_ct_) and diffusion resistance (*R*_s_), respectively.^[^ ^64^, ^65^ ^]^ The R_ct_ value of the Al@S/AB@CC‐CoS_2_ is 108 Ω, which is smaller than those of the Al@S/AB@CC (117 Ω) and Al@S/AB (140 Ω). The Nyquist plots of the cells recorded after 10 cycles at 0.5 C were also performed as shown in Figure S4, Supporting Information. It is obvious that all plots have an additional semicircle in the high frequency region which corresponds to the interfacial resistance (*R*_f_) resulted from the irreversible Li_2_S_2_/Li_2_S layer.^[^ ^66^ ^]^ R_f_ value of Al@S/AB@CC‐CoS_2_ cell (1.5 Ω) is smaller than that of Al@S/AB@CC cell (2.7 Ω). The Al@S/AB@CC‐CoS_2_ cell after 10 cycles delivers a significantly decreased *R*_ct_ (6.8 Ω), thus indicating improved electrolyte infiltration upon cycling. Compared with the Al@S/AB@CC cell, the Al@S/AB@CC‐CoS_2_ cell delivers smaller R_ct_ values before and after cycling, suggesting that the CC‐CoS_2_ film contributes to faster charge transfer kinetics during the polysulfides conversion. These electrochemical results reveal the CC‐CoS_2_ film can work as an excellent barrier to block polysulfide shuttle effect, and the polar CoS_2_ nanoparticles have high catalytic activity to accelerate the polysulfide conversion reactions.

**Figure 4:**
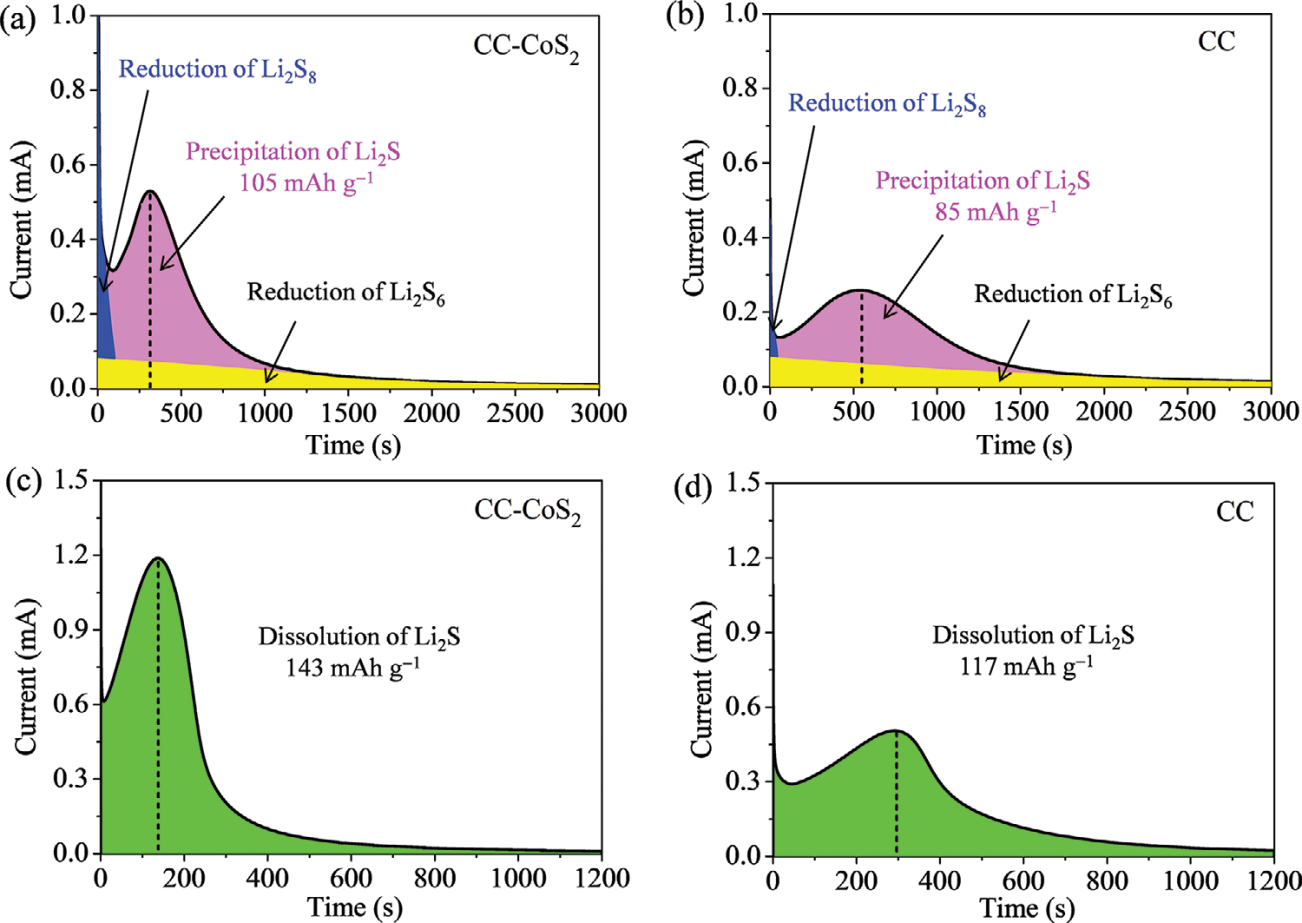
a,b) Potentiostatic discharge curves of the Li_2_S_8_ catholyte at 2.02 V on surfaces of a) CC‐CoS_2_ and b) CC. The blue/yellow colors indicate the reduction of Li_2_S_8_/Li_2_S_6_, while the pink color represents the precipitation of Li_2_S. c,d) Potentiostatic charge curves at 2.40 V to evaluate the dissolution kinetics of Li_2_S on surface of c) CC‐CoS_2_ and d) CC.

The Li_2_S precipitation test was carried out on surfaces of CC‐CoS_2_ and CC hosts with addition of 0.1 m Li_2_S_8_ catholyte (20 µL) as the active material. The two cells were potentiostatically discharged at 2.02 V for 50 min, after they were galvanostatically discharged to 2.06 V. **Figure** **4**a,b shows the time‐dependent discharge current profiles of Li_2_S deposition on the CC‐CoS_2_ and CC surfaces, respectively. The deposition of Li_2_S on CC‐CoS_2_ surface is much faster with a higher peak current than on CC. The CC‐CoS_2_ cell reaches the peak current of 0.53 mA with a time of 310 s, while the CC cell requires 537 s to achieve the peak current of 0.26 mA. Furthermore, the CC‐CoS_2_ cell yields a precipitation capacity of 105 mAh g^−1^, which is much larger than that (85 mAh g^−1^) of the CC cell. This observation reveals the CC‐CoS_2_ has the better catalytic activity, which enables the more efficient utilization of lithium polysulfides.^[^ ^67^, ^68^, ^69^ ^]^ The potentiostatic Li_2_S dissolution tests were also performed (Figure 4c,d). The CC‐CoS_2_ cell exhibits a much larger dissolution capacity (143 mAh g^−1^) than the CC cell (117 mAh g^−1^). All these results further reveal that the CoS_2_ nanoparticles covered on CC surface have a superior electrocatalytic activity to promote the deposition and dissolution of Li_2_S.

Cycling performance of the Al@S/AB@CC‐CoS_2_, Al@S/AB@CC and Al@S/AB cells were evaluated. Figure 3j displays the cycling performance of the three cells at 0.5 C rate. The specific capacity of the Al@S/AB@CC‐CoS_2_ cell stabilizes at 1150 mA h g^−1^ after 100 cycles at 0.5 C. In a distinct contrast, the Al@S/AB@CC and Al@S/AB cells yield respectively lower reversible specific capacities of 777 and 271 mAh g^−1^ after 100 cycles. Cycle stability of the Al@S/AB@CC‐CoS_2_ cell was further investigated by analyzing the evolution of Q_L_/Q_H_ ratio at representative cycles (Figure S5, Supporting Information). Q_H_ (Q_L_) is 408 (847) mAh g^−1^ at the 2nd cycle, and slightly decreases to 399 (834) mAh g^−1^ at 20th cycle, to 378 (815) mAh g^−1^ at 60th cycle, and to 352 (793) mAh g^−1^ at 100th cycle, corresponding to a capacity retention of 98% (98%), 93% (96%), and 86% (94%), respectively. The high retention rates indicate that the shuttle effect has been effectively suppressed. Importantly, Q_L_/Q_H_ ratio increases from 2.08 (2nd) to 2.09 (20th), 2.16 (60th), and 2.25 (100th). The increase in Q_L_/Q_H_ ratio indicates improved catalytic conversion of polysulfides during cycling process, which is responsible for the well‐preserved capacity.

After cycling for 100 cycles at 0.5 C, the three cells were disassembled to observe color change of the diaphragms, owing to soluble polysulfide diffusion. The separator of the Al@S/AB cell becomes yellowish obviously, while the Al@S/AB@CC‐CoS_2_ and Al@S/AB@CC cells show a separator with little color change (Figure S6, Supporting Information). This observation also reveals the shuttle effect of polysulfides is significantly suppressed and loss of active material is effectively prevented by incorporating CC‐CoS_2_ barrier in LSBs. XPS spectra of the CC‐CoS_2_ after cycling were recorded to gain a better understanding of the chemical adsorption and catalytic effect of CoS_2_ on polysulfides. The XPS peaks of Co 2p exhibit a slight shift to lower binding energy (794.0 eV→793.3 eV, 778.9 eV→778.6 eV) relative to those before cycling, suggesting the Co─S*_n_* ^2−^ interaction (Figure S8a, Supporting Information). The S 2p peak at 162.7 eV corresponding CoS_2_ also shows a shift of 0.2 eV to lower binding energy (Figure S8b, Supporting Information). These shifts are considered to be resulted from the interaction between CoS_2_ nanoparticles and negatively charged polysulfides.^[^ ^61^ ^]^ The polythionate (168.8 eV), thiosulfate (167.1 eV), Li_2_S_4_ (163.8 eV), and Li_2_S_2_ (161.8 eV) are also detected from the S 2p spectrum as shown in Figure S8b, Supporting Information. These peaks can be ascribed to the catalytic reactions of long‐chain polysulfides with CoS_2_ to form intermediate thiosulfate and short‐chain polysulfides.^[^ ^70^ ^]^

To further investigate the synergistic effect of physical blocking, chemical absorption and catalytic conversion, a long‐term cycling test of the Al@S/AB@CC‐CoS_2_ cell was carried out with a high current rate of 4.0 C. As shown in Figure 3k, the Al@S/AB@CC‐CoS_2_ cell displays a discharge capacity of 775 mAh g^−1^ after the activation at 0.2 C for the initial three cycles. The capacity increases to 874 mAh g^−1^ after following 200 cycles and then remains at 610 mAh g^−1^ after 1000 cycles. The average decay rate of specific capacity is only 0.021% per cycle at 4 C rate, thereby indicating the excellent operation stability of the Al@S/AB@CC‐CoS_2_ cell. Therefore, the Al@S/AB@CC‐CoS_2_ cell with a low sulfur loading is demonstrated with superior electrochemical performance as indicated by the high specific capacity, excellent rate capacity, and unexpected long‐term cycling stability.

**Figure 5:**
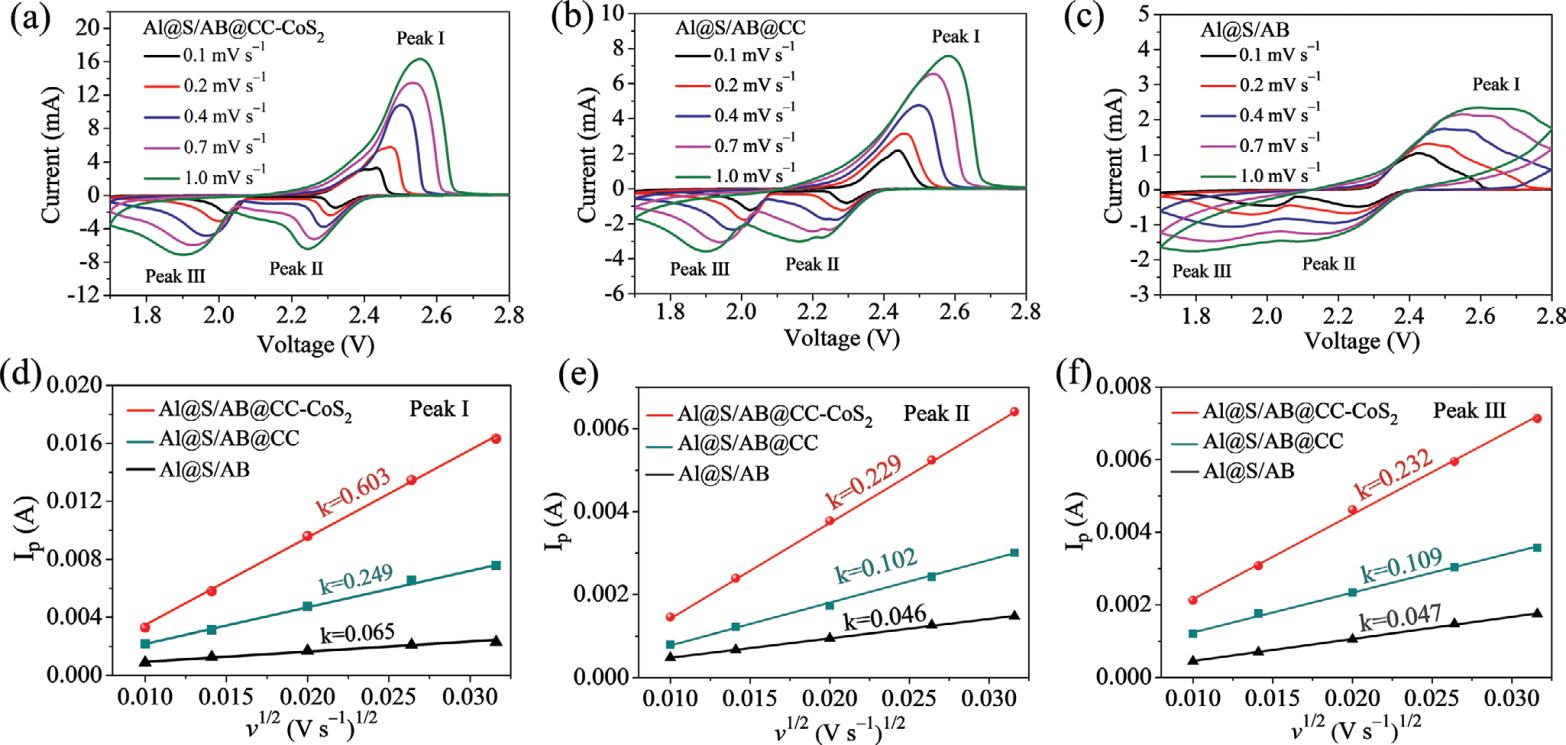
a–c) CV curves of a) Al@S/AB@CC‐CoS_2_, b) Al@S/AB@CC, and c) Al@S/AB cells at various scanning rates. d–f) The corresponding relationship between peak currents and scanning rates of the three cells.

CV measurements at various scan rates from 0.1 to 1.0 mV s^−1^ were conducted on Al@S/AB@CC‐CoS_2_, Al@S/AB@CC, and Al@S/AB cells to study the Li^+^ diffusion coefficients (**Figure** **5**a–c). The peak current of the oxidation peak and two reduction peaks at different potentials increases with the accelerated scan rate for the three cells. Figure 5d–f shows the linear curves obtained by fitting the peak current related to the scanning rate, according to the Randles–Sevcik equation:^[^ ^59^, ^71^ ^]^

(1) $$I_{p}=2.69\times 10^{5}\cdot n^{3/2}\cdot A\cdot {D_{\mathrm{Li}}}^{1/2}\cdot C_{\mathrm{Li}}\cdot v^{1/2}$$

where *I*_p_ is the peak current, *n* represents the number of transferred electrons (*n* = 2 in LSB), *A* stands for the electrode area (≈1.13 cm^2^), *D*_Li_ corresponds to the diffusion coefficient of Li^+^ (cm^2^ s^−1^), *v* indicates the scan rate, and *C*_Li_ means the Li^+^ concentration in the electrolyte (mol cm^−3^). Therefore, *D*_Li_ values can be evaluated based on the slopes of the fitted curves. The Al@S/AB@CC‐CoS_2_ cell has the largest slopes of k = 0.603 for peak I, k = 0.229 for peak II, k = 0.232 for peak III. The *D*_Li_ values are calculated to be 38.2 × 10^−10^, 5.51 × 10^−10^, and 5.64 × 10^−10^ cm^2^ s^−1^ for peaks I, II, and III, respectively. These values are nearly 5–6 times higher than those of Al@S/AB@CC cell (6.05 × 10^−10^, 1.09 × 10^−10^, and 1.25 × 10^−10^ cm^2^ s^−1^). The Al@S/AB cell has the lowest D_Li_ values (0.44 × 10^−10^, 0.22 × 10^−10^, and 0.23 × 10^−10^ cm^2^ s^−1^). These data mean that the Li^+^ diffusion rate is significantly accelerated for the Al@S/AB@CC‐CoS_2_ cell by incorporating the CC‐CoS_2_ film. The accelerated *D*_Li_ values of the Al@S/AB@CC‐CoS_2_ cell were also proved by galvanostatic intermittent titration technique (GITT) as shown in Figure S7, Supporting Information. The 3D porous CC‐CoS_2_ film can selectively sieve Li^+^ ions while effectively suppress undesired polysulfides migration to the anode side. The fast Li^+^ diffusion at various stages in the Al@S/AB@CC‐CoS_2_ cell indicates outstanding redox reaction kinetics during polysulfide conversion.

**Figure 6:**
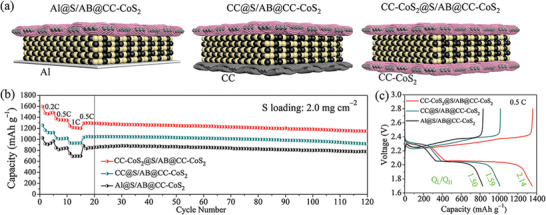
a) Schematic diagrams of three cells of Al@S/AB@CC‐CoS_2_, CC@S/AB@CC‐CoS_2_, and CC‐CoS_2_@S/AB@CC‐CoS_2_. b) Rate performance of the three cells with a higher sulfur loading. c) Galvanostatic discharge/charge profiles of the three cells at 0.5 C.

Besides working as an excellent barrier, the CC‐CoS_2_ film can also serve as a promising 3D current collector in LSBs with a higher sulfur loading. To highlight the advantages of CC‐CoS_2_ as a current collector in buffering volume expansion, promoting catalytic conversion reaction and slowing shuttle effect, electrochemical performances of LSBs using CC‐CoS_2_, CC, and Al foil as current collectors were compared. **Figure** **6**a shows the schematic diagram of three different structure cells of Al@S/AB@CC‐CoS_2_, CC@S/AB@CC‐CoS_2_, and CC‐CoS_2_@S/AB@CC‐CoS_2_. The rate capabilities of Al@S/AB@CC‐CoS_2_, CC@S/AB@CC‐CoS_2_, and CC‐CoS_2_@S/AB@CC‐CoS_2_ cells with a sulfur loading of 2.0 mg cm^−2^ were evaluated by cycling at different current rates from 0.2 to 1 C (Figure 6b). Significantly, the CC‐CoS_2_@S/AB@CC‐CoS_2_ cell yields high reversible specific capacities of 1471, 1353, and 1209 mAh g^−1^ at the current rates of 0.2, 0.5, and 1 C. After the rate performance test, the CC‐CoS_2_@S/AB@CC‐CoS_2_ cell maintains a high specific capacity 1150 mAh g^−1^ at 0.5 C after following 100 cycles, indicating good cycling stability. In comparison, the rate performances of both CC@S/AB@CC‐CoS_2_ and Al@S/AB@CC‐CoS_2_ cells at all rates are much lower than those of CC‐CoS_2_@S/AB@CC‐CoS_2_ cell. Figure 6c shows the galvanostatic charge/discharge profiles of the three cells at 0.5 C. The capacities contributed by the higher‐discharge plateau (Q_H_) and the lower‐discharge plateau (Q_L_) are clearly revealed. The Al@S/AB@CC‐CoS_2_ cell has a relatively low Q_H_ and Q_L_ values of 332 and 497 mAh g^−1^, corresponding to a Q_L_/Q_H_ ratio of 1.50. The Q_L_/Q_H_ ratio slightly increases to 1.59 when CC was used as the current collector (CC@S/AB@CC‐CoS_2_), and the discharge capacity increases to 1011 mAh g^−1^. Furthermore, the CC‐CoS_2_@S/AB@CC‐CoS_2_ cell exhibits the longest discharge platform (Q_L_ = 916 mAh g^−1^) and the highest Q_L_/Q_H_ ratio of 2.14. The enhancement in both Q_H_ and Q_L_ indicates the better capture of polysulfide species at the surface of 3D CC‐CoS_2_. The increased Q_L_/Q_H_ ratio is attributed to improved catalytic conversion kinetics of polysulfides by the active CoS_2_ catalyst on the CC‐CoS_2_ current collector. These results confirm that the CC‐CoS_2_ current collector plays a key role in improving the electrochemical performance of LSBs with a high sulfur loading. The symmetrical structure where the active material is sandwiched between two CC‐CoS_2_ films (i.e., CC‐CoS_2_@S/AB@CC‐CoS_2_) is promising to achieve decent performance for high sulfur loading LSBs.

**Figure 7:**
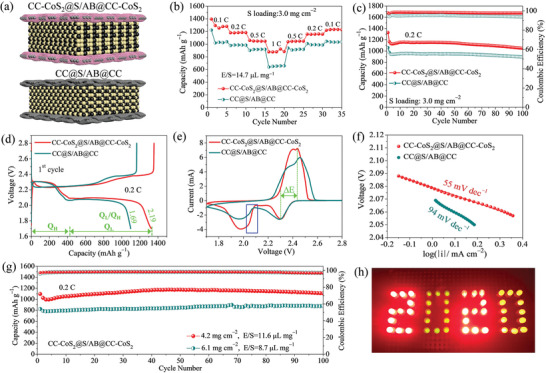
a) Schematic diagrams of two symmetrically sandwiched cells of CC‐CoS_2_@S/AB@CC‐CoS_2_ and CC@S/AB@CC. b) Rate performance, c) cycling performance at 0.2 C, d) the initial discharge/charge profiles at 0.2 C, e) CV curves scanned at a rate of 0.1 mV s^−1^, f) the corresponding Tafel plots of the two cells of CC‐CoS_2_@S/AB@CC‐CoS_2_ and CC@S/AB@CC with a sulfur loading density of 3.0 mg cm^−2^. g) Cyclic performance of the CC‐CoS_2_@S/AB@CC‐CoS_2_ cell with sulfur loading densities of 4.2 and 6.1 mg cm^−2^ at 0.2 C. h) Photographs of 46 LED lamps lighted by the CC‐CoS_2_@S/AB@CC‐CoS_2_ cell.

Owing to the advantages of efficient physical blocking, strong chemisorption, high catalytic activity, fast Li^+^ kinetics, high conductivity, and 3D porous structure, the CC‐CoS_2_ film is attractive to be applied to high sulfur loading LSBs. The electrochemical performance of the CC‐CoS_2_@S/AB@CC‐CoS_2_ cell with high sulfur loadings of 3.0–6.1 mg cm^−2^ was further investigated. **Figure** **7**a shows the schematic diagram of symmetrically sandwiched structure of the CC‐CoS_2_@S/AB@CC‐CoS_2_ and the CC@S/AB@CC as a control sample. The rate performance of the two symmetrical cells with a sulfur loading of 3.0 mg cm^−2^ is presented in Figure 7b. The CC‐CoS_2_@S/AB@CC‐CoS_2_ cell yields a higher initial specific capacity than the CC@S/AB@CC cell at 0.1 C. The average reversible specific capacities of the CC‐CoS_2_@S/AB@CC‐CoS_2_ cell are estimated to be 1278, 1181, 1048, and 878 mAh g^−1^ at 0.1, 0.2, 0.5, and 1 C, respectively. The specific capacity can be well recovered to 1045 mAh g^−1^ at 0.5 C, to 1156 mAh g^−1^ at 0.2 C, and to 1232 mAh g^−1^ at 0.1 C as the discharge current decreases. As comparison, the CC@S/AB@CC cell exhibits lower specific capacities of 1029, 986, 916, and 653 mAh g^−1^ at 0.1, 0.2, 0.5, and 1 C, respectively. The two cells with a sulfur loading of 3.0 mg cm^−2^ were cycled at 0.2 C (Figure 7c). The CC‐CoS_2_@S/AB@CC‐CoS_2_ cell shows initial discharge/charge capacities of 1328/1357 mAh g^−1^ with a coulombic efficiency of 97.86%, which is higher than that (94.46%) of the CC@S/AB@CC cell. After 100 cycles, the CC‐CoS_2_@S/AB@CC‐CoS_2_ cell maintains a discharge capacity of 1049 mAh g^−1^ with a coulombic efficiency of 97.68%, while the CC@S/AB@CC cell keeps a capacity of 894 mAh g^−1^ with a coulombic efficiency of 93.74%. Improved capacities and coulombic efficiencies are proved for the CC‐CoS_2_@S/AB@CC‐CoS_2_ cell. The initial discharge/charge profiles of the two cells at 0.2 C are shown in Figure 7d. It is clear that the CC‐CoS_2_@S/AB@CC‐CoS_2_ cell has a Q_L_ value of 912 mAh g^−1^ and a Q_L_/Q_H_ ratio of 2.19, which are better than those of the CC@S/AB@CC cell (Q_L_ = 691 mAh g^−1^, Q_L_/Q_H_ = 1.69). This scenario indicates that CC‐CoS_2_@S/AB@CC‐CoS_2_ cell can stabilize more polysulfides in the cell region, and have a higher sulfur utilization efficiency as well as a better catalytic conversion capability compared with the CC@S/AB@CC cell. Figure 7e displays CV curves of the two cells scanned at a rate of 0.1 mV s^−1^. Higher peak current and narrower separation (ΔE) between redox peaks are observed for the CC‐CoS_2_@S/AB@CC‐CoS_2_ cell, supporting its superior catalytic activity. Moreover, it also exhibits a lower cathodic Tafel slope (55 mV dec^−1^) derived from the polarization curve in the range of 2.04–2.10 V (Figure 7f) and smaller charge transfer resistances before and after cycling (Figure S9, Supporting Information). These observations further confirm that the CC‐CoS_2_@S/AB@CC‐CoS_2_ cell has better catalytic activity, accelerated redox kinetics, and improved electrochemical performance owing to incorporation of the CoS_2_ catalyst in the symmetrically sandwiched structure. Because of these merits, electrochemical performance of the CC‐CoS_2_@S/AB@CC‐CoS_2_ cell with even higher sulfur loading of 4.2 and 6.1 mg cm^−2^ was investigated (Figure 7g). High initial discharge capacities of 1097 and 823 mAh g^−1^ at 0.2 C are obtained for the cell with sulfur loading of 4.2 and 6.1 mg cm^−2^, respectively. Importantly, no capacity loss is observed after 100 cycles for both cells (1106 mAh g^−1^ at the sulfur loading of 4.2 mg cm^−2^ and 885 mAh g^−1^ at the sulfur loading of 6.1 mg cm^−2^). High capacity retention over 100% after 100 cycles are achieved for both cells (Figure S10, Supporting Information). The performance is among the best of high sulfur loading LSBs as shown in Table S1, Supporting Information. The CC‐CoS_2_@S/AB@CC‐CoS_2_ cell can light up 46 light‐emitting diode (LED) lamps at the same time (Figure 7h), showing a great potential for practical applications.

**Figure 8:**
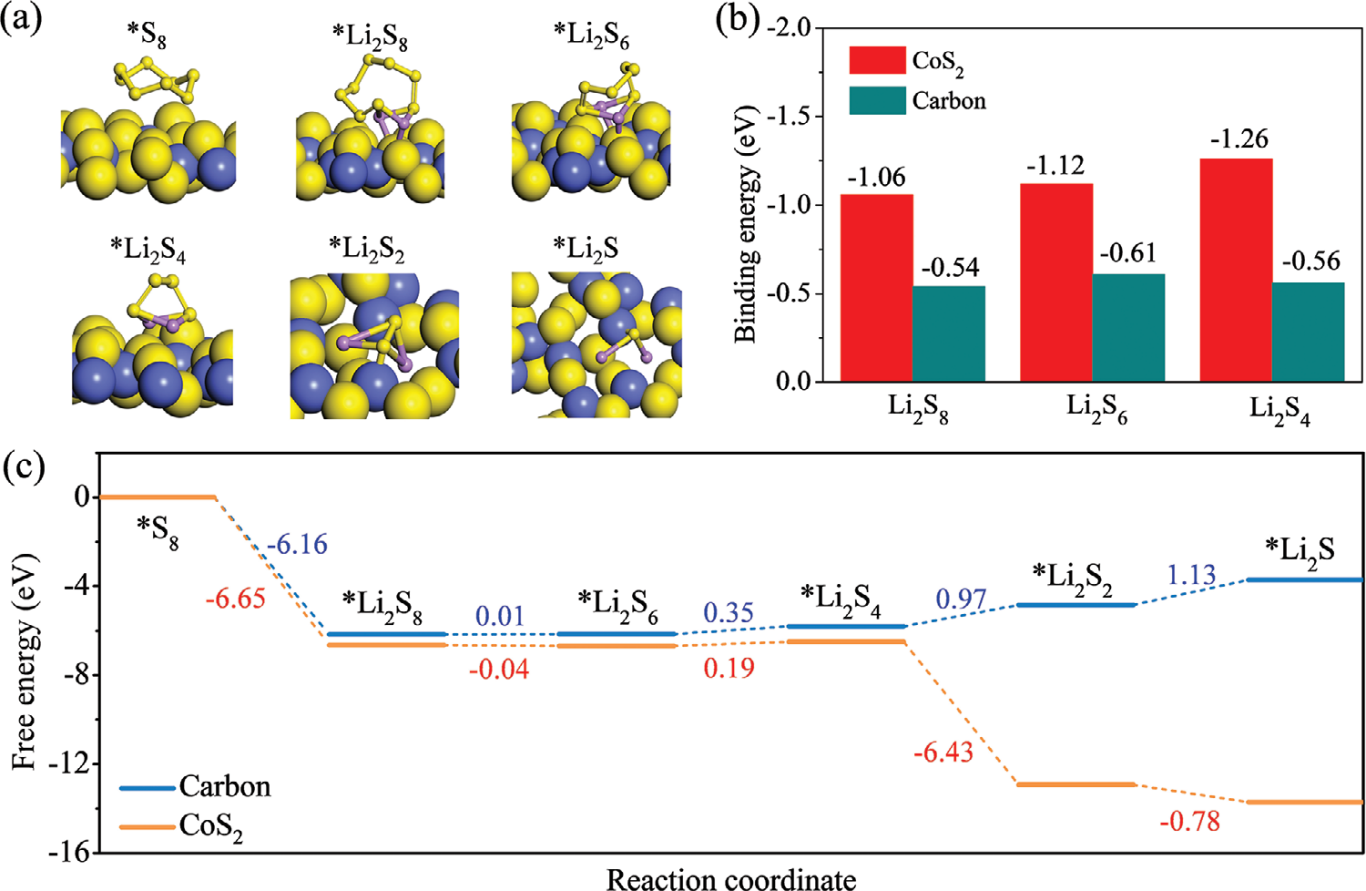
a) The optimized adsorption configurations of intermediate species on CoS_2_ (111) plane. b) Comparison of binding energies of the soluble lithium polysufides with CoS_2_ and carbon surface. c) Free energy profiles for the reduction of Li_2_S*_n_* on catalytic surfaces of CoS_2_ and carbon, respectively.

Density functional theory (DFT) calculation was conducted to investigate the binding energies of lithium polysulfides on CoS_2_ and CC surfaces. The optimized structures of the various lithium polysulfides and S_8_ molecules are shown in Figure S11, Supporting Information. The adsorption configurations of these sulfur species on the CoS_2_ (111) plane and the carbon basal plane are presented in **Figure** **8**a and Figure S12, Supporting Information, respectively. The binding energies of soluble polysulfides (Li_2_S*_n_*, *n* = 4, 6, and 8) on CoS_2_ are −1.26, −1.12, and −1.06 eV, which are much higher than those on carbon (Figure 8b). The higher binding energy indicates the stronger chemical anchoring of soluble Li_2_S*_n_*.^[^ ^61^, ^72^ ^]^ The calculations match well with the visual adsorption test in Figure 2a, confirming the improved adsorption capability by coating CoS_2_ on CC. The charge density of soluble Li_2_S*_n_* absorbed on CoS_2_ and carbon are quite different owing to their diverse electrostatic affinities. A pronounced charge accumulation and redistribution between Li_2_S*_n_* and CoS_2_ substrate occurs. Abundant electrons transfer to the Li─S bond, producing stable adsorption of Li_2_S*_n_* on CoS_2_ at a molecular level. On the contrary, less electron distribution is found on carbon surface, which results in weak atomic interaction and inferior binding energy of Li_2_S*_n_* on carbon. In the discharge process, it involves the reduction of S_8_ with Li^+^ to form the various intermediate lithium polysulfides of Li_2_S_8_, Li_2_S_6_, Li_2_S_4_, and Li_2_S_2_, and the final product of Li_2_S. The Gibbs free energies were evaluated for the above reactions on CoS_2_ and carbon surfaces as displayed in Figure 8c. According to the calculated Gibbs free energy changes, the reaction from S_8_ to Li_2_S_8_ is spontaneous and the conversion from Li_2_S_8_ to Li_2_S_6_/Li_2_S_4_ is nearly thermodynamic equilibrium on both CoS_2_ and carbon surfaces. The reduction of Li_2_S_4_ to Li_2_S_2_/Li_2_S has the obviously positive free energy changes (0.97 and 1.13 eV) on carbon, implying that this conversion of Li_2_S_2_ to Li_2_S is the rate‐controlling step in the discharge process. In contrast, the same transformation happens spontaneously with large negative free energy changes (−6.43 and −0.78 eV) on CoS_2_ surface. These calculations indicate that the reduction of polysulfides species is more thermodynamically favorable on CoS_2_ surface than that on carbon surface.

## 3 Conclusions

In summary, we reported an effective strategy to fabricate high‐performance LSBs by using the as‐prepared CC‐CoS_2_ film as both current collector and physicochemical barrier for capturing soluble lithium polysulfides and promoting their redox reactions. The CC‐CoS_2_ has the advantages of 3D porous structure, high conductivity, strong chemisorption, high catalytic activity, efficient physical blocking, and fast Li^+^ transport kinetics. Theoretical calculations reveal that catalytic reduction of sulfides species is thermodynamically more favorable on CoS_2_ surface than on carbon. Compared with the pristine CC, the CC‐CoS_2_ exhibits improved physical confinement, enhanced chemical adsorption, and accelerated redox reaction kinetics of polysulfide species. Benefitting from these merits, the LSBs assembled from CC‐CoS_2_ exhibit significant improvements in discharge capacity, rate capability, and cycling stability. The discharge capacity at 1 C increases from 200 mAh g^−1^ for Al@S/AB to 700 mAh g^−1^ for Al@S/AB@CC and 1159 mAh g^−1^ for Al@S/AB@CC‐CoS_2_. The Al@S/AB@CC‐CoS_2_ cell also shows high rate capability (≈823 mAh g^−1^ at 4 C) and long cycling stability. Furthermore, the symmetrically sandwiched cathode of CC‐CoS_2_@S/AB@CC‐CoS_2_ is beneficial for high sulfur loading LSBs. The CC‐CoS_2_@S/AB@CC‐CoS_2_ cell with a sulfur loading of 3.0 mg cm^−2^ has initial discharge/charge capacities of 1328/1357 mAh g^−1^ with a coulombic efficiency of 97.86% at 0.2 C, which are much better than those of the CC@S/AB@CC cell. Significantly, no capacity loss is observed for the CC‐CoS_2_@S/AB@CC‐CoS_2_ cells with high sulfur loadings of 4.2 and 6.1 mg cm^−2^ after 100 cycles at 0.2 C, indicating their excellent cycling stability. This symmetrically sandwiched cathode based on high catalytic metal chalcogenides modified CC opens up a new door for developing high‐performance LSBs with a high sulfur loading.

## 4 Experimental Section

### Materials

Cobaltous sulfate (CoSO_4_·7H_2_O, AR 99.5%), sublimed sulfur (S, AR 99.5%), AB, N‐Methyl‐2‐pyrrolidone (NMP, 99.5 wt%), polyvinylidence fluoride (PVDF, 99.5 wt%), lithium sulfide (Li_2_S, AR 99.9%), lithium bis (trifluoromethanesulfonyl) imide (LiTFSI, 99.95 wt%) and lithium nitrate (LiNO_3_, 99.99 wt%) were purchased from Aladdin. Carbon cloth (CC, HCP330N) was purchased from HESEN. 1,3‐dioxolane (DOL, AR 99.5%), dimethyl ether (DME, AR 99.5%), and thiourea (CS(NH_2_)_2_, AR 99%) were obtained from Macklin.

### Synthesis of the CC‐CoS_2_

The CC‐CoS_2_ was obtained using a one‐step hydrothermal synthesis. A carbon cloth (2 × 2 cm^−2^) was pre‐treated in hydrochloric acid (≈37%) for 1 h and then washed with deionized (DI) water. For the synthesis, 0.6 mmol of CoSO_4_·7H_2_O and 1.8 mmol of CS(NH_2_)_2_ were dissolved in 35 mL DI water under vigorous stirring to form a homogeneous solution, and then the solution was transferred to a 50 mL Teflon‐lined stainless steel autoclave, then the cleaned carbon cloth (2 × 2 cm^−2^) was immersed in the solution and keep at 180 °C for 24 h. After cooling down to ambient temperature, the carbon cloth covered with CoS_2_ nanoparticles was obtained and washed with DI water and dried at 60 °C for 12 h.

### Characterization

XRD patterns were obtained from an X‐Pert PRO MPD diffractometer with a Cu K*α* radiation. SEM images were recorded on a field‐emission SEM (Philips XL30 FEG). TEM and HRTEM images were obtained from a JEM‐2100F electron microscope at an accelerating voltage of 200 kV. XPS measurement was carried out by using a VG ESCALAB 220i‐XL UHV system with an Al K*a* X‐ray source (1486.6 eV). Raman spectra were recorded on a Lab RAM HR spectroscope with a 532 nm excitation. The UV–vis analysis was obtained from an ultraviolet visible near infrared spectrophotometer (CARY 5000) to evaluate the adsorption capability of different anchor materials.

### Preparation of Sulfur Electrodes

An electrode slurry was made by mixing 60 wt% sublimed sulfur, 30 wt% AB, and 10 wt% PVDF in NMP. The slurry was then coated onto the CC‐CoS_2_ and dried at 60 °C for 12 h under vacuum to obtain the CC‐CoS_2_@S/AB electrode. The sulfur loading density of the CC‐CoS_2_@S/AB electrode is tunable in the range of 2–6 mg cm^−2^. The slurry was coated onto the Al foil to obtain the Al@S/AB electrode with a sulfur loading of ≈1.2 mg cm^−2^. These sulfur electrodes were punched into disks with 12.0 mm in diameter for assembling coin cells.

### Fabrication of Coin Cells

The CR2032 cells were assembled in an Ar‐filled glove box (H_2_O<0.1 ppm and O_2_<0.1 ppm). The Li foil (diameter of 15.6 mm) and the Celgard 2400 sheets (diameter of 16.0 mm) were used as the anode and the separator, respectively. The electrolyte was prepared by dissolving 1 m LITFSI in a mixed solution of DOL and DME (1:1 in volume) with addition of 1 wt% LiNO_3_. The CC‐CoS_2_@S/AB@CC‐CoS_2_ battery was assembled by covering the CC‐CoS_2_ on the CC‐CoS_2_@S/AB electrode as the cathode, while the Al@S/AB@CC‐CoS_2_ battery was fabricated by covering the CC‐CoS_2_ on the Al@S/AB electrode as the cathode. The parameters of areal sulfur loading, electrolyte amount, and electrolyte/sulfur ratio in each cell were listed in Table S2, Supporting Information.

### Fabrication of the CC‐CoS_2_||CC‐CoS_2_ and the CC||CC Symmetrical Cells

Symmetrical cells were fabricated to verify catalytic effect of the pristine CC and the CC‐CoS_2_. The pristine CC or the CC‐CoS_2_ was cut into circular disks with a diameter of 12 mm. CR2032 coin cells were assembled in an Ar‐filled glove box by using two identical electrodes, a Celgard 2400 separator, and 40 µL electrolyte of 1 m LiTFSI and 0.2 m Li_2_S_6_ in 1:1 (v/v) DOL/DME.

### Electrochemical Measurements

The electrochemical performances of the various samples were measured by assembling CR2032 coin cells. Galvanostatic discharge/charge tests and GITT were carried out on a NewareBTS2300 system (Shenzhen, China) at 25 °C in voltage range of 1.7 to 2.8 V. CV measurements scanned at rates from 0.1 to 1 mV s^−1^ and EIS measurements conducted from 100 kHz to 0.1 Hz with potentiostatic amplitude of 5 mV were carried out on a CHI660E (Shanghai, China) electrochemical workstation.

### Lithium Polysulfide Adsorption Tests

The Li_2_S_6_ solution (2 mmol L^−1^) was prepared by stoichiometric amounts of sulfur and lithium sulfide (Li_2_S) with a molar ratio of 5:1 dissolving in DOL/DME (1:1 in volume) mixture and stirring at room temperature in an Ar‐filled glove box. The CC‐CoS_2_ (24 mg) and the pristine CC (20 mg) were added in the Li_2_S_6_ solution (4 mL), respectively. The adsorption capability of the CC‐CoS_2_ and the CC to Li_2_S_6_ was investigated by UV–vis spectroscopy.

### Theoretical Calculations

All calculations were carried out by spin‐polarized DFT as implemented in Vienna Ab initio Simulation Package (VASP) 6.1.1 with Perdew–Burke–Ernzerhof generalized gradient approximation.^[^ ^73^, ^74^ ^]^ A supercell of graphene containing 5 × 5 unit cells was employed to model carbon system. As for CoS_2_ system, 2 × 2 unit cells of (111) plane with terminated sulfur atoms was used. The cutoff energy was set as 420 eV after cutoff testing and the k‐points were set to be 3 × 3 × 1 for geometry optimization for CoS_2_ and carbon optimization. The electronic energy and forces were converged to within 1 × 10^−5^ eV and 0.02 eV Å^−1^, respectively. For charge density and frequency calculations, the energy was converged to within 10^−6^ and 10^−7^ eV, respectively. The van der Waals interactions were considered by the method of the Grimme (DFT+D3). The effect of water was taken into consideration using VASP implicit solvent model.^[^ ^75^ ^]^ For quantitatively measuring the interaction between the substrates and Li_2_S*_n_*, we defined the binding energy E_b_ as follows:

(2) $$E_{b}=E\left(\mathrm{Total}\right)-E\left(\mathrm{Sub}\right)-E\left(Li_{2}S_{n}\right)$$

where E(Sub), E(Li_2_S*_n_*), and E(Total) represent the total energies of the substrate, the Li_2_S*_n_* cluster, and the adsorption pair of the substrate and Li_2_S*_n_*, respectively.

## Conflict of Interest

The authors declare no conflict of interest.

## Supporting information

Supporting Information

## Data Availability

Research data are not shared.
