# Supplementary material for: Sandwiched Cathodes Assembled from CoS2‐Modified Carbon Clothes for High‐Performance Lithium‐Sulfur Batteries
Source: Adv Sci (Weinh). 2021 Jun 2;8(16):2101019. doi: 10.1002/advs.202101019 (PMC8373102; doi:10.1002/advs.202101019)
Supplement: Supplementary file 1 — Supporting Information [file ADVS-8-2101019-s001.pdf]

© 2021 Wiley-VCH GmbH

ADVANCED SCIENCE
Open Access

Supporting Information

for *Adv. Sci.*, DOI: 10.1002/advs.202101019

Sandwiched Cathodes Assembled from CoS_2-Modefied Carbon Cloth for High-Performance Lithium-Sulfur Batteries

Jun Xu, Likun Yang, Shoufu Cao, Jingwen Wang, Yuanming Ma, Junjun Zhang,* Xiaoqing Lu**

Supporting Information

Sandwiched Cathodes Assembled from CoS_2-Modified Carbon Cloth for High-Performance Lithium-Sulfur Batteries

Jun Xu, Likun Yang, Shoufu Cao, Jingwen Wang, Yuanming Ma, Junjun Zhang,* Xiaoqing Lu**

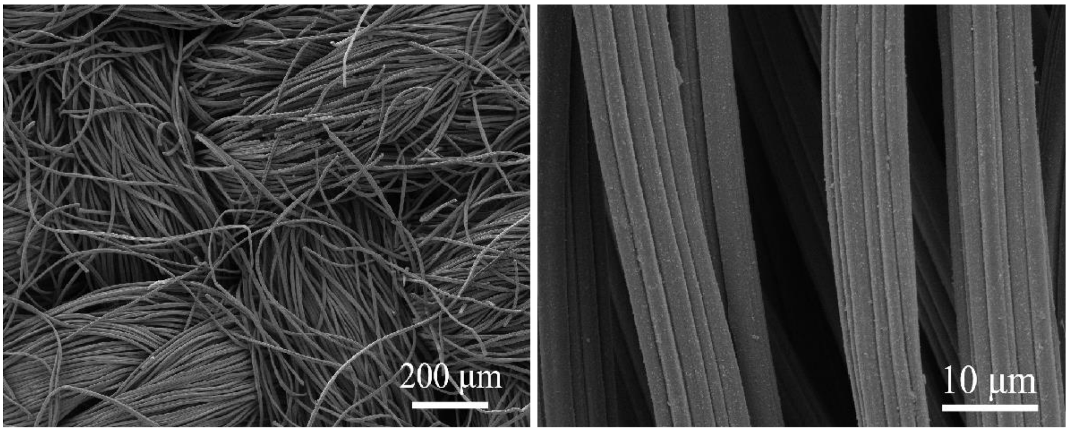

Figure S1. SEM images of CC.

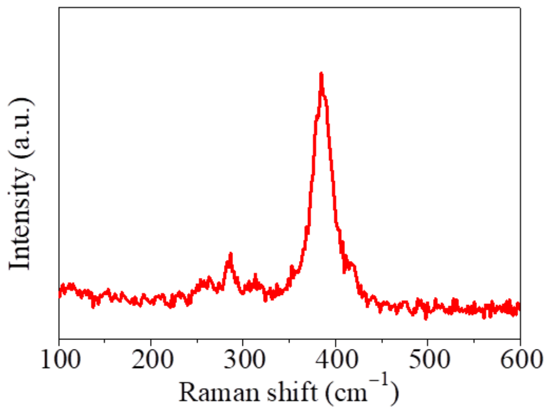

Figure S2. Raman spectrum of the CC-CoS_2.

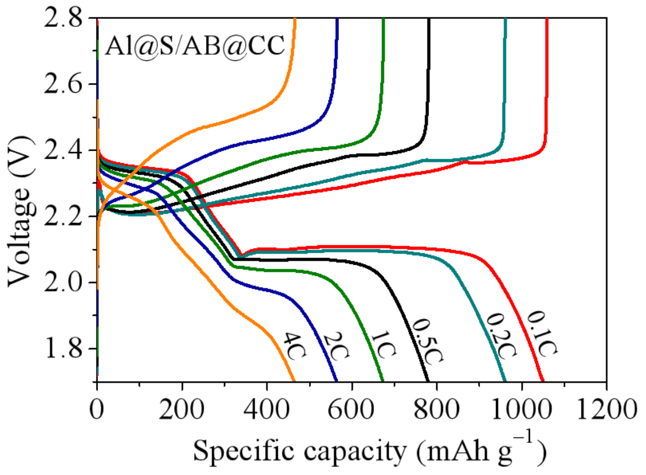

Figure S3. Galvanostatic charge-discharge voltage profiles of the Al@S/AB@CC cell at different rates from 0.1 to 4C.

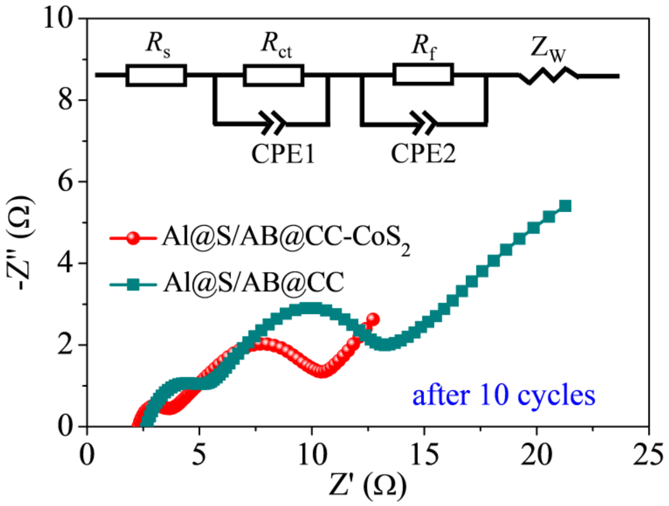

Figure S4. Nyquist plots of the Al@S/AB@CC-CoS_2 and Al@S/AB@CC cells after 10 cycles at 0.5C, inset is the corresponding equivalent circuit.

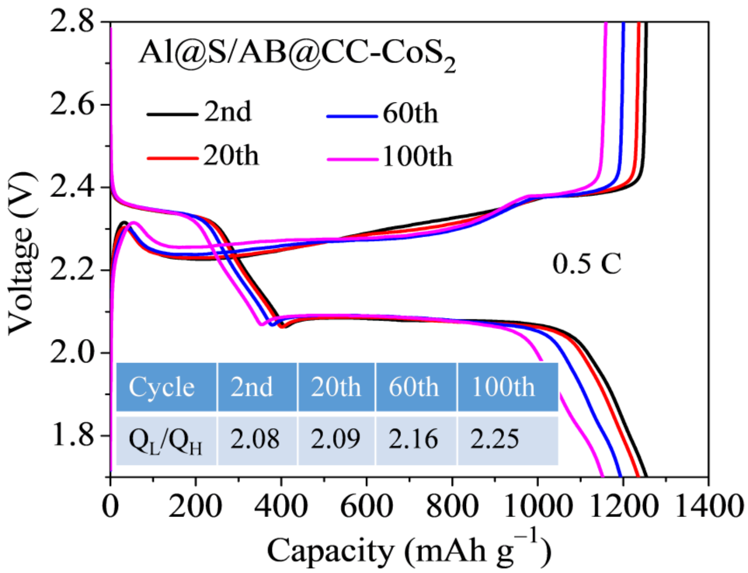

Figure S5. Representative charge/discharge profiles of the Al@S/AB@CC-CoS_2 cell at 0.5 C.

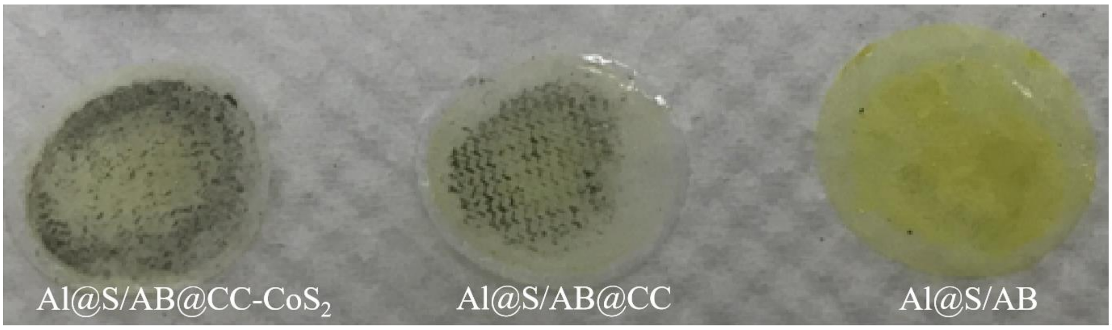

Figure S6. Photograph of various separators obtained by disassembling the coin cells without further washing step. Each cell was operated at 0.5C rate for 100 cycles.

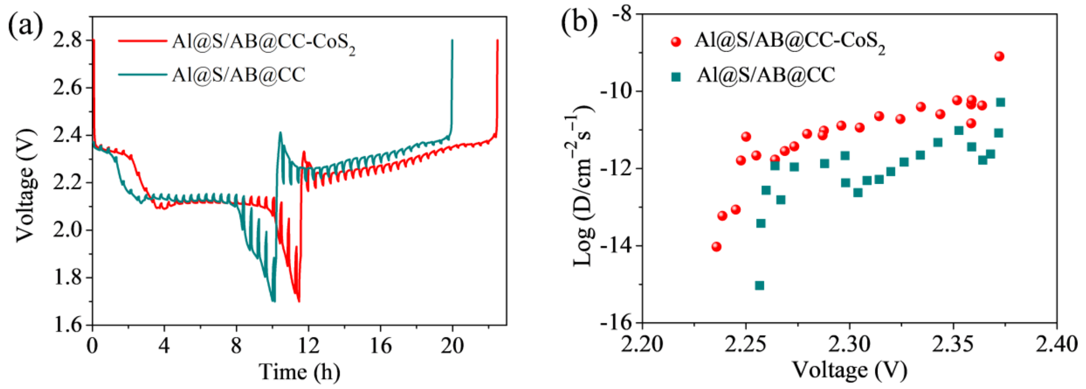

Figure S7. (a) GITT curves of the Al@S/AB@CC-CoS_2 and Al@S/AB@CC cells. The profiles were collected with a current pulse at 0.1C for 20 min and followed with 5 min rest. (b) Various D_{Li} at the charge state of the two cells.

Galvanostatic intermittent titration technique (GITT) was also employed to investigate the Li^+ diffusion coefficients (D_{Li}) of the Al@S/AB@CC-CoS_2 and Al@S/AB@CC cells at different states. **Figure S7a** shows the potential response profiles of the two cells. The discharge/charge plateaus of the Al@S/AB@CC-CoS_2 and Al@S/AB@CC cells in the GITT curves can approximately match with the typical discharge/charge profiles.

The D_{Li} value can be obtained via the following Equation 1,

$$D_{\mathrm{Li}} = \frac{4}{\pi\tau}\left(\frac{m_A V_m}{M_A S}\right)^2 \left(\frac{\Delta E_s}{\Delta E_\tau}\right)^2 \qquad (1)$$

where τ (s) is the constant current pulse time, m_A (g), M_A (g mol^{-1}), V_m (cm^3 mol^{-1}) are the mass, molar weight and molar volume of the active material, respectively, S is the surface area of the electrode (1.13 cm^2), ΔE_s is the voltage difference in the steady state potential of the step at plateau, ΔE_τ is the voltage change during a constant current pulse time excluding the iR drop.

Figure S7b presents the D_{Li} values of the Al@S/AB@CC-CoS_2 and Al@S/AB@CC cells at charge state calculated from each pulse. Obviously, the Al@S/AB@CC-CoS_2 cell exhibits higher D_{Li} values than the Al@S/AB@CC cell over the delithiation state.

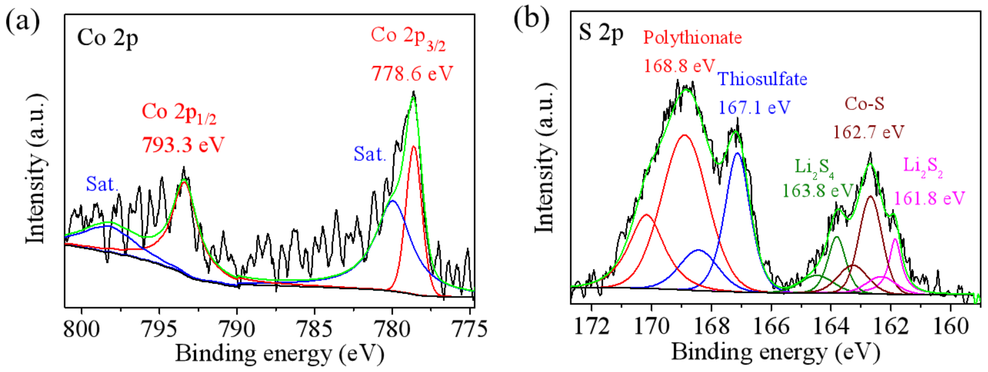

Figure S8. XPS spectra of Co 2p (a) and S 2p (b) after 100 cycles at 0.5C.

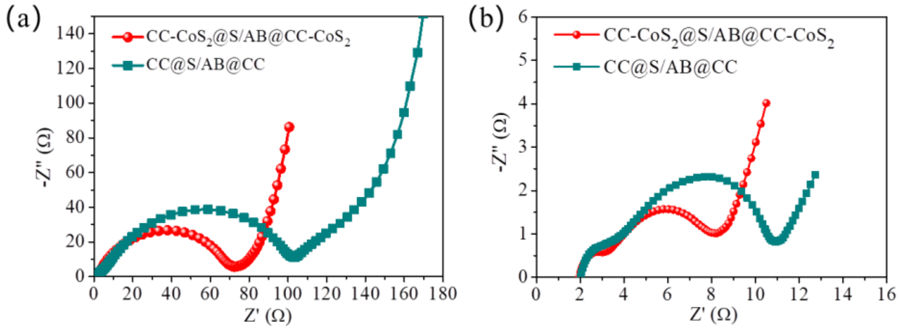

Figure S9. Nyquist plots of the two cells (CC-CoS_2@S/AB@CC-CoS_2 and CC@S/AB@CC): (a) before cycling and (b) after 5 cycles at 0.2 C.

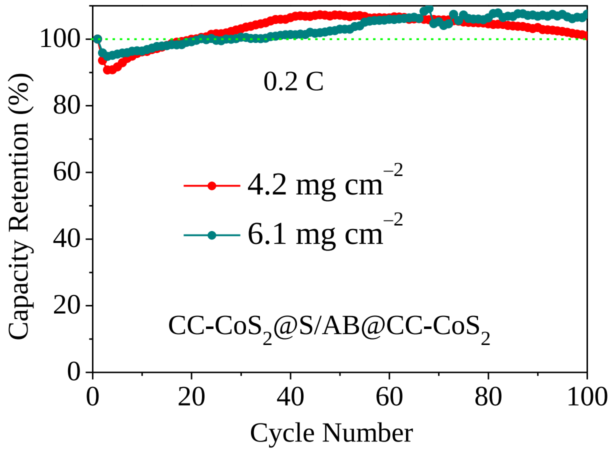

Figure S10. Capacity retention of the CC-CoS_2@S/AB@CC-CoS_2 cells with high sulfur loadings of 4.2 and 6.1 mg cm^{-2}.

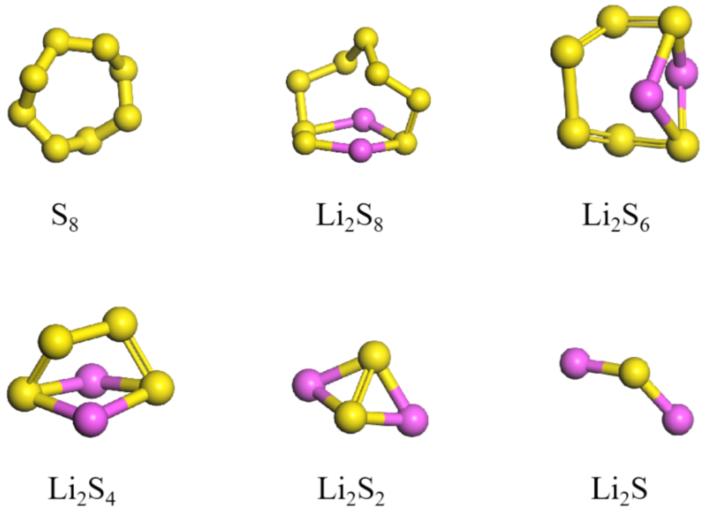

Figure S11. Optimized structures of S_8 and Li_2S_n molecules, n = 1, 2, 4, 6, 8.

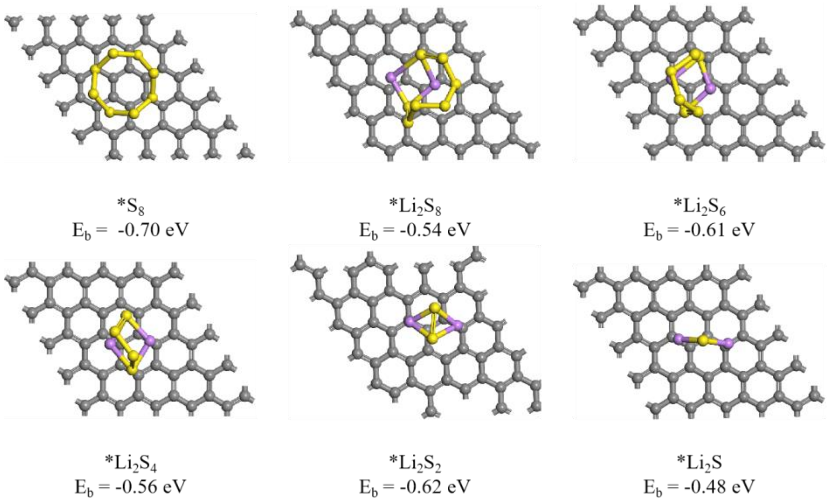

Figure S12. Binding energy and adsorption configurations of Li_2S_n (n = 1, 2, 4, 6, 8) on carbon substrate.

Table S1. Recent advances of electrochemical performance of LSBs with various current collectors and interlayer barriers.

Current Collector	Cathode	Interlayer/ Separator	Sulfur loading (mg cm^{-2})	Cycle Number/ Cycled Capacity (mAh g^{-1}) / Rate (C)	Decay Per Cycle (%)	References
Al	S/AB	CC-CoS_2	1.2	100/1050/0.5	0.21	Our work
				1000/610/4	0.021	
CC-CoS_2	S/AB	CC-CoS_2	4.2	100/1106/0.2	no decay	
			6.1	100/885/0.2	no decay	
Al	S/HPGC/Super P	CoS_2/HPGC	3.0	250/846/0.2	0.079	Small **2020**, 16, 2002046
Al	S/Super P	Co_9S_{8-X}/CNT	2	1000/648.5/0.3	0.049	ACS Nano **2019**, 13, 7073−7082
Carbon-coated Al foil	S/Super P	MoS_2@CMT	2	100/1069/0.5	0.085	Adv. Sci. **2020**, 7, 1903260
Al	S/Carbon black	C-C-N-Co	1	100/787/0.5		Small Methods **2020**, 4, 1900701
			4.1	100/3.1 mAh cm^{-2}/0.5		
Al	S/Super P	NOCNF	4.54	100/908/0.1	0.041	ACS Appl. Energy Mater. **2019**, 2, 777−787
Al	S/AB	3D MoS_2	1.2	100/941/0.5	0.14	ACS Appl. Energy Mater. **2019**, 2, 1702−1711
CoS_2@ NGCNs	CoS_2@NGCNs/S		1.3±0.2	300/519.4/1	0.075	Adv. Funct. Mater. **2019**, 29, 1903712
CoS_2-SPAN-CNT	CoS_2-SPAN-CNT		2.4	100/1204/0.2	0.31	J. Mater. Chem. A. **2020**, 8, 1298
C@$NiSe_2$	C@$NiSe_2$/S		5	200/1030/0.2	0.089	J. Mater. Chem. A. **2019**, 7, 15302
			12	100/680/0.1		
CC@CoP/C	CC@CoP/C-S		1.81	100/1257/0.1		Adv. Mater. **2019**, 31, 1902228
			3.21	100/944/0.1		
C@WS_2	C@WS_2/S		1-1.2	200/1000/0.5	0.08	Adv. Energy Mater. **2017**, 7, 1601843
CNF@Co_3S_4	S/CNF@Co_3S_4		1.7	200/694.4/1	0.011	Adv. Sci. **2020**, 7, 2002037
			6.8	100/592/0.1		
Al	Co_9S_8/MWCNTs/S		1.5	400/549/0.5	0.087	J. Energy Chem. **2020**, 46, 22–29

Al	GCS1-S		1.5	500/460/0.5	0.11	J. Energy Chem. **2020**, 48, 109–115
Al	S@CoS_2-LBLCN		1.8	1000/463.3/5	0.033	Adv. Sci. **2019**, 6, 1802362
			3	300/793.5/0.5		
Al	S/uCoS_2@MMC		3	100/833.2/0.2	0.013	J. Mater. Chem. A, **2020**, 8, 10885

Table S2. The parameters of the various LSBs.

Cells	Sulfur mass (mg)	Areal sulfur loading (mg cm^{-2})	Electrolyte amount (μL)	E/S ratio (μL mg^{-1})
Al@S/AB@CC-CoS_2	1.36	1.2	30	22.1
CC-CoS_2@S/AB@CC-CoS_2	3.39	3.0	50	14.7
	4.75	4.2	55	11.6
	6.89	6.1	60	8.7
